# Sarcopenia Risk in Tenerife: Prevalence, Multidimensional Vulnerability, and the Socio-Economic Case for Prevention and Treatment

**DOI:** 10.3390/diseases14050175

**Published:** 2026-05-18

**Authors:** Vicente Llinares Arvelo, Carlos Enrique Martinez Alberto, David González-Martín, Serafin Corral

**Affiliations:** 1 Escuela de Enfermería Nuestra Señora de la Candelaria, Campus of Enfermery, 38200 Santa Cruz de Tenerife, Spain; vllinare@ull.edu.es (V.L.A.); extcmartina@ull.edu.es (C.E.M.A.); 2 Faculty of Medicine, European University Miguel de Cervantes, 47012 Valladolid, Spain; 3 Department of Applied Economics and Quantitative Methods, University of La Laguna, 38200 Santa Cruz de Tenerife, Spain

**Keywords:** sarcopenia, SARC-F, frailty, prevalence, cost-effectiveness, economic burden, Tenerife, Canary Islands, primary care, healthy ageing

## Abstract

**Background/Objectives:** Sarcopenia—the progressive loss of skeletal muscle mass and function—is a growing public health challenge in ageing populations. Island territories face compounded vulnerabilities due to distinct epidemiological and socio-economic profiles. This study examines sarcopenia risk prevalence among community-dwelling older adults in Tenerife (Canary Islands, Spain) and estimates the economic burden alongside the cost-effectiveness of evidence-based interventions. **Methods:** A cross-sectional study was conducted among 374 community-dwelling older adults (mean age 80.4 years, SD 4.8; 51.1% female) recruited from primary care health centres across three health zones in Tenerife. Participants were stratified into a control group without established chronic disease-related functional decline (Group 1; *n* = 274) and a case group with multimorbidity and functional limitations (Group 3; *n* = 100). Sarcopenia risk was assessed using the SARC-F questionnaire (threshold ≥ 4). A comprehensive geriatric battery—including the Barthel Index, FRAIL scale, MNA-SF, Pfeiffer test, SPPB, handgrip dynamometry, and IPAQ—characterised multidimensional vulnerability. Annual direct and indirect costs were estimated using unit costs from Spanish national health accounts, and intervention cost-effectiveness was modelled using published meta-analytic data. **Results:** Overall sarcopenia risk prevalence was 36.4% (*n* = 136; SARC-F ≥ 4), rising to 83.0% in the case group versus 19.3% in controls (OR ≈ 21.5, *p* < 0.001). Prevalence was 42.1% in males and 30.9% in females. Diabetes was independently associated with elevated risk (44.8% vs. 29.9%; OR 1.90, 95% CI 1.23–2.92; *p* = 0.003). Health Zone 1 exhibited the highest prevalence (63.0%) versus Zones 2 (23.5%) and 3 (32.8%). Multidimensional vulnerability was pervasive: 28.6% of participants were frail, 75.7% had nutritional compromise, 11.5% showed moderate cognitive impairment, and 89.8% reported low or no physical activity. The estimated annual socio-economic cost of sarcopenia in Tenerife is approximately EUR 88.9 million (Spain nationally: EUR 12.1 billion). Combined exercise–nutrition interventions yield cost-per-QALY ratios of EUR 3800–7000, far below Spain’s EUR 25,000/QALY threshold. **Conclusions:** Sarcopenia constitutes a major, multidimensionally compounded health burden in Tenerife’s older population, concentrated among frail, diabetic, nutritionally compromised, and physically inactive individuals. The economic case for universal SARC-F screening and multicomponent intervention is compelling, exceeding cost-effectiveness thresholds by a wide margin. Territorial disparities in burden call for equity-oriented, place-based resource allocation within the Canarian health system.

## 1. Introduction

Sarcopenia—operationally defined by reduced muscle mass combined with low muscle strength and/or diminished physical performance—has been increasingly recognised as one of the defining geriatric syndromes of the twenty-first century [1,2]. First formally described by Rosenberg [3] and subsequently standardised by the European Working Group on Sarcopenia in Older People (EWGSOP2; Cruz-Jentoft et al. [1]), the condition affects an estimated 10–27% of community-dwelling older adults globally, with prevalence rising sharply beyond the age of 80. A recent systematic review and meta-analysis of 35 studies estimated global sarcopenia prevalence to be 10.0% in community-dwelling adults [4]. In Spain, national epidemiological surveys—including the ELES (Longitudinal Study on Ageing in Spain) cohort—estimate prevalence between 14.7% and 33.6% depending on diagnostic criteria, setting, and age structure [5].

Ageing island populations present particular epidemiological and socio-economic challenges that are not fully captured by mainland-calibrated models. The Canary Islands, classified as an ultraperipheral region under Article 349 of the Treaty on the Functioning of the European Union (TFEU), combine an ageing demographic structure, structural dependence on tourism, geographic isolation, and a fragmented territorial health infrastructure [6]. Tenerife—the most populous island of the archipelago, with approximately 930,000 inhabitants—is thus a critical site for understanding how sarcopenia intersects with social vulnerability, health system capacity, and economic sustainability. Despite this context, island-specific sarcopenia prevalence data and subnational economic burden estimates for ultraperipheral regions such as the Canary Islands remain virtually absent from the published literature—a gap that constrains evidence-based health planning and perpetuates the systematic under-allocation of preventive resources to peripheral territories.

Beyond its clinical dimensions, sarcopenia imposes a substantial socio-economic burden through multiple pathways: elevated rates of falls and fractures leading to hospital admission, progressive functional dependence and institutionalisation, increased demand for informal caregiving, heightened use of primary and specialist care, and premature mortality [7,8]. Global estimates of the annual economic burden of sarcopenia range from USD 18.5 billion in the United States [9] to over EUR 50 billion across the European Union [10], though subnational and island-specific estimates remain poorly characterised.

The socio-economic case for sarcopenia prevention is equally compelling from a cost-effectiveness standpoint. Evidence from randomised controlled trials and meta-analyses consistently demonstrates that resistance exercise training, protein supplementation, and combined multicomponent interventions can reduce sarcopenia incidence by 25–55%, with cost-per-quality-adjusted-life-year (QALY) ratios far below standard willingness-to-pay thresholds [11,12]. Yet these interventions remain systematically under-deployed in primary care settings, particularly in peripheral and island territories where health service provision is structurally constrained [6].

This study addresses four interrelated objectives: (i) to characterise the prevalence of sarcopenia risk in community-dwelling older adults in Tenerife using the validated SARC-F screening tool, stratified by sex, age group, comorbidity, and health zone; (ii) to map the multidimensional vulnerability profile of the affected population; (iii) to estimate the direct and indirect economic costs of sarcopenia at island and national levels; and (iv) to evaluate the cost-effectiveness of available prevention and treatment strategies and derive policy implications for the Canarian and Spanish health systems.

## 2. Materials and Methods

### 2.1. Study Design and Setting

This cross-sectional epidemiological study was conducted in Tenerife, Canary Islands, Spain, across nine primary care health centres of the Servicio Canario de Salud distributed throughout the island, encompassing areas in the north and south of Tenerife and including both metropolitan and rural settings so as to capture the territorial and sociodemographic heterogeneity of the older population. The study was conducted within the Doctoral Programme in Health Sciences (Programa de Doctorado en Ciencias de la Salud) at the University of La Laguna, following methodological recommendations for observational epidemiological studies and international guidelines for clinical research in older populations. Data collection was carried out between November 2024 and December 2025.

### 2.2. Participants

A total of 374 community-dwelling older adults aged 75 years and above were enrolled. Health-centre selection followed a non-probabilistic procedure based on the availability and voluntary collaboration of nursing staff at each centre. Within each participating centre, eligible patients aged ≥75 years attending their scheduled primary care appointments during the data-collection period were consecutively invited to participate; the overall response rate among eligible individuals approached was approximately 87%. Participants were classified into two analytical groups: Group 1 (control; *n* = 274), comprising individuals without a prior diagnosis of chronic disease-related functional decline, and Group 3 (case; *n* = 100), comprising individuals with established multimorbidity and functional limitations. For analytical purposes, participants were classified into two clinical profile groups on the basis of their recorded multimorbidity and functional status: Group 1 (control; individuals without chronic disease-related functional decline) and Group 3 (case; individuals with established multimorbidity and functional limitations). This stratification enabled comparison of sarcopenia risk prevalence across contrasting functional profiles within the same primary care population. Exclusion criteria included acute illness at time of assessment, cognitive impairment severe enough to preclude informed consent, and inability to complete the physical assessment battery. Because centre selection was non-probabilistic, the representativeness of the sample with respect to the full primary care population of Tenerife cannot be fully guaranteed; this constraint is addressed under Limitations (Section 4.6). The mean age of the total sample was 80.4 years (SD 4.8; range: 75–97 years); 51.1% were female. Body mass index (BMI) was calculated as weight in kilograms divided by height in metres squared; the mean BMI was 24.7 kg/m^2^.

### 2.3. Sarcopenia Screening: SARC-F Questionnaire

Sarcopenia assessment followed the hierarchical algorithm proposed by EWGSOP2 [1], which distinguishes between probable, confirmed, and severe sarcopenia based on sequential evaluation of muscle strength, mass, and physical performance. The process was initiated with the SARC-F questionnaire [13], a validated five-item self-report screening tool (Strength, Assistance with walking, Rising from a chair, Climbing stairs, Falls; each item scored on a scale of 0–2; maximum 10 points). A score ≥ 4 indicated high probability of sarcopenia and triggered the subsequent steps of the algorithm. In participants screening positive, muscle strength was assessed by bilateral handgrip dynamometry using the EWGSOP2 sex-specific cut-offs for low strength (men: <27 kg; women: <16 kg), enabling identification of probable sarcopenia. Physical performance was then assessed with the Short Physical Performance Battery (SPPB [14]; see Section 2.4); an SPPB score ≤ 8 in combination with low grip strength was used to classify severe sarcopenia. As dual-energy X-ray absorptiometry (DXA) and bioimpedance analysis (BIA) were not available in this primary care setting, demonstration of low muscle mass—required for confirmed sarcopenia per EWGSOP2—was not feasible. All sarcopenia estimates therefore correspond to probable sarcopenia (SARC-F ≥ 4 with or without low grip strength) and are described throughout as “sarcopenia risk” to reflect this diagnostic limitation consistently.

### 2.4. Complementary Clinical Assessments

A multi-domain geriatric assessment battery was administered to all participants. Frailty was evaluated using the FRAIL scale [15] (five components: fatigue, resistance, ambulation, illness, and loss of weight; scores: 0 = robust, 1–2 = pre-frail, ≥3 = frail). The FRAIL scale is conceptually distinct from the Fried Frailty Phenotype [16]: whereas the Fried criteria require objective performance measures (grip-strength dynamometry and timed gait speed) alongside self-reported exhaustion and weight loss, the FRAIL scale is a fully self-reported, five-item questionnaire specifically designed for rapid primary care screening without the need for physical performance testing. Nutritional status was assessed with the Mini Nutritional Assessment Short-Form (MNA-SF [17], scored as follows: 12–14 = normal, 8–11 = at nutritional risk, 0–7 = malnourished). Functional independence was measured with the Barthel Index [18] (0–100; categorised as independent ≥90, mild dependence 60–89, moderate dependence 40–59, and severe dependence <40). Cognitive function was evaluated using the Pfeiffer Short Portable Mental Status Questionnaire [19] (0–2 errors = no impairment, 3–4 = mild, 5–7 = moderate, ≥8 = severe). Physical performance was measured with the Short Physical Performance Battery (SPPB [14]; 0–12, four performance categories). Grip strength was measured bilaterally using a hand-held dynamometer, with sex-specific cut-offs consistent with the EWGSOP2 criteria [1] (women: <16 kg; men: <27 kg). Physical activity was assessed using the short form of the International Physical Activity Questionnaire (IPAQ [20]), yielding metabolic equivalent (MET)-minutes per week and categorical activity levels (inactive, low, moderate, and high).

### 2.5. Economic Cost Estimation

Direct healthcare costs attributable to sarcopenia were estimated by multiplying sarcopenia-attributable healthcare utilisation rates (from published systematic reviews and Spanish health administrative data) by unit costs derived from the Ministerio de Sanidad’s 2022 health accounts [21] and IMSERSO’s 2022 long-term care expenditure report [22]. Indirect costs—including informal care burden and productivity losses among working-age caregivers—were estimated using replacement cost and human capital methodologies, respectively. Tenerife-specific extrapolations applied island-level population denominators for the over-75 age group (approximately 65,000 individuals; INE 2023 [23]). All monetary values are expressed in 2023 euros.

### 2.6. Cost-Effectiveness Modelling

Intervention cost-effectiveness was assessed using published meta-analytic data on the efficacy and per-patient costs of seven categories of sarcopenia prevention and treatment. Cost-per-QALY ratios were estimated using a simplified decision-analytic framework assuming a 5-year time horizon, a 3% annual discount rate, and utility weights derived from EQ-5D-based mapping from sarcopenia severity categories [10,11]. Interventions were graded as follows: A (≤EUR 10,000/QALY, strong evidence), B (EUR 10,001–20,000/QALY, moderate evidence), or C (EUR 20,001–25,000/QALY, limited evidence).

### 2.7. Statistical Analysis

Descriptive statistics are reported as frequencies and percentages for categorical variables, and means with standard deviations for continuous variables. Between-group comparisons used chi-squared tests for categorical variables and independent-samples *t*-tests or Mann–Whitney U tests for continuous variables as appropriate. Odds ratios with 95% confidence intervals were calculated for key risk factors. Variables were included in the binary logistic regression model a priori on the basis of theoretical relevance and established epidemiological association with sarcopenia risk (diabetes status, sex, age group, and health zone); stepwise selection was not used, minimising inflation of type-I error. Binary logistic regression was therefore used to estimate adjusted ORs with 95% CIs for each pre-specified predictor simultaneously. All analyses were conducted in R (version 4.3.1; R Core Team, Vienna, Austria [24]). Where expected cell counts fell below 5 in chi-squared analyses, exact *p*-values were obtained via Monte Carlo permutation correction (10,000 simulations). Effect sizes were quantified using the phi coefficient (φ) for associations between pairs of dichotomous variables and Cohen’s d for between-group comparisons of continuous variables, expressed as mean (SD). Statistical significance was set at *p* < 0.05.

## 3. Results

### 3.1. Participant Characteristics

Table 1 presents the socio-demographic and clinical characteristics of the 374 participants. The case group (Group 3) was significantly older than the controls (82.3 vs. 79.7 years, *p* < 0.001) and had a higher proportion of males (58.0% vs. 45.6%, *p* = 0.045). Diabetes mellitus was present in 43.6% of all participants. Mean BMI was 24.7 kg/m^2^. Multidimensional vulnerability was pervasive: 71.4% were classified as frail, 56.6% had nutritional compromise, 89.8% reported low or no physical activity, and 54.5% exhibited moderate-to-severe functional dependence according to the Barthel Index.

### 3.2. Sarcopenia Prevalence: Overall and Subgroup Analyses

Overall, 136 participants (36.4%) screened positive for sarcopenia risk (SARC-F ≥4). The distribution of SARC-F total scores showed progressive accumulation at higher score ranges; 34.3% of all participants scored ≥6 points. Prevalence differed markedly across analytical subgroups (Table 2).

The most striking finding was the divergence between study groups: 83.0% of case participants (Group 3) exceeded the sarcopenia risk threshold compared with only 19.3% of controls (Group 1), yielding a crude odds ratio of approximately 21.5 (*p* < 0.001). Within the case group, Group 3 males had the highest prevalence at 87.9% (51/58), while Group 3 females showed 76.2% (32/42); the corresponding figures in the control group were 20.8% (26/125) and 18.1% (27/149) for males and females, respectively. Across the overall sample, prevalence was higher among males (42.1%) than females (30.9%), with grip strength dynamometry confirming that 63.9% of men and 24.1% of women exhibited low grip strength.

Diabetes mellitus was associated with significantly elevated sarcopenia risk (44.8% vs. 29.9%; OR 1.90, 95% CI 1.23–2.92; *p* = 0.003). Territorial heterogeneity was pronounced: Health Zone 1 exhibited a prevalence of 63.0%, compared with 23.5% in Zone 2 and 32.8% in Zone 3. Age-stratified analysis showed broadly consistent prevalence across age bands—34.4% in the 71–75-year group, 36.1% in participants aged 76–80 years, 38.1% in participants aged 81–85 years, 34.8% in participants aged 86–90 years, and 42.9% in participants aged ≥91 years (*n* = 14), though small cell sizes limit interpretation in the oldest group.

### 3.3. Multidimensional Vulnerability Profile

Frailty co-occurrence was near-universal: 71.4% of participants were classified as frail (FRAIL score ≥ 3), 27.0% as pre-frail, and only 1.6% as robust. Nutritional compromise was widespread: 64.3% screened positive for malnutrition risk on MNA-SF (score 8–11) and a further 11% were malnourished (score 0–7); only 64.7% had at risk of malnutrition. Intact intellectual functioning was present in 88.1% (Pfeiffer 0–2 errors), with clinical significance for adherence to self-managed exercise and dietary prescriptions. Physical inactivity was the most prevalent modifiable risk factor: 89.8% of participants fell below recommended physical activity thresholds, with 4.5% completely sedentary. The SPPB revealed that 12.6% (*n* = 47) showed severe physical limitation (Category 1) and 26.2% (*n* = 98) showed moderate limitation (Category 2), together indicating that 38.8% of participants had clinically meaningful gait, balance, and lower-limb strength impairments.

### 3.4. Economic Burden of Sarcopenia in Tenerife

Applying the study’s overall prevalence estimate (36.4%) to Tenerife’s population aged ≥75 years (approximately 65,000 individuals; INE 2023 [23]), we estimate that approximately 23,660 individuals are currently living with sarcopenia risk in Tenerife. Table 3 presents the estimated annual economic burden. The total annual socio-economic cost reaches approximately EUR 88.9 million for the island and EUR 12.1 billion nationally. Direct costs (primary care, hospitalisations, rehabilitation, nutritional supplementation, and long-term care) account for approximately 55.7% of the total burden (EUR 49.5 million in Tenerife). Indirect costs (informal care burden and productivity losses) contribute a further 44.3% (EUR 39.3 million). Long-term care and institutionalisation represents the single largest cost category (EUR 31.0 million in Tenerife; EUR 4.20 billion nationally). Applying a conservative 60% reducibility estimate consistent with multicomponent intervention evidence [25], approximately EUR 53.3 million annually in Tenerife could theoretically be averted through widespread evidence-based prevention.

### 3.5. Cost-Effectiveness of Prevention and Treatment

Table 4 summarises the cost-effectiveness profiles of eight intervention categories. Multicomponent exercise–nutrition programmes demonstrated the most favourable cost-per-QALY ratios (EUR 3800–7000), with break-even horizons of one to two years. Primary care-based SARC-F screening (EUR 18–35 per screen) achieved cost-per-QALY ratios below EUR 2800. Resistance exercise training alone (EUR 480–820/patient/year) yielded ratios of EUR 4200–8500/QALY, while protein supplementation programmes (EUR 290–550/year) achieved EUR 5100–9200/QALY. All Grade A interventions fall substantially below Spain’s conventional cost-effectiveness threshold of EUR 25,000/QALY.

## 4. Discussion

### 4.1. Prevalence in Context

The 36.4% overall sarcopenia risk prevalence observed in this Tenerife sample substantially exceeds estimates from most comparable European community-based studies. Population-level SARC-F-based estimates from the SHARE study range from 7.3% to 27.4% across European nations [28], while Spanish-specific estimates from the ELES cohort approximate 25–30% in the over-75 age group [5]. Our higher estimate likely reflects the composition of our sample—including a case group with established multimorbidity—as well as the advanced mean age of 80.4 years and structural socio-economic characteristics of the Canarian population. The 83.0% prevalence in Group 3 is particularly striking and consistent with the known relationship between multimorbidity burden and sarcopenia risk, forming a self-reinforcing cycle of malnutrition, physical inactivity, metabolic dysregulation, and functional decline.

### 4.2. Territorial Inequalities and the Health Zone Effect

The three-fold difference in sarcopenia risk prevalence between Health Zone 1 (63.0%) and Zone 2 (23.5%) is one of the study’s most policy-relevant findings. This territorial gradient reflects the well-documented relationship between place-based socio-economic deprivation and accelerated biological ageing [29], operating through cumulative disadvantages in nutrition quality, access to physical activity infrastructure, and healthcare utilisation. Within the Canarian health system, Zone 1 health centres typically serve urban-peripheral or rural populations with lower median income and more limited access to community exercise facilities, underscoring the need for place-based health equity interventions.

### 4.3. The Compound Geriatric Vulnerability Nexus

A distinctive contribution of this study is its simultaneous documentation of sarcopenia risk within a multi-domain vulnerability profile. The constellation observed—sarcopenia risk (36.4%), frailty (71.4%), nutritional compromise (56.6%), cognitive impairment (58.8%), physical inactivity (89.8%), and functional dependence (54.5%)—constitutes a mutually reinforcing compound geriatric vulnerability nexus with significant implications for clinical management and economic modelling. Standard cost-of-illness models that treat sarcopenia as a single-disease entity underestimate the full economic burden by failing to account for interaction effects that compound care needs and accelerate institutionalisation. The 58.8% prevalence of moderate cognitive impairment is particularly important: individuals with cognitive limitations require supervised rather than self-managed exercise and dietary prescriptions, substantially increasing per-patient costs and constraining intervention reach.

### 4.4. The Economic Case for Prevention

The economic argument for sarcopenia prevention is robust across multiple analytical frameworks. From a health economics perspective, all Grade A interventions identified meet cost-effectiveness thresholds by a wide margin, with break-even horizons of one to two years. The estimated EUR 53.3 million annual preventable burden in Tenerife alone—against an investment requirement of perhaps EUR 10–15 million for broad intervention coverage—implies a return on investment of three to five times within a five-year window. These findings align with European multicomponent intervention programmes, such as SPRINTT [26], and with evidence-based advocacy analyses demonstrating high returns from prevention investment [27]. The long-term care and informal care burden estimated here (EUR 53 million combined in Tenerife) also represents a form of socio-economic cost systematically underestimated in GDP-based accounting, predominantly borne by female family members outside formal healthcare expenditure tracking.

### 4.5. Policy Implications

Five concrete policy recommendations emerge: (i) universal SARC-F screening should be integrated into annual over-75 health checks in the Canarian primary care system at an annual cost of approximately EUR 1.2–2.3 million—less than 3% of the estimated annual sarcopenia burden; (ii) multicomponent exercise–nutrition programmes should be systematically commissioned through Zone 1 health centres with subsidised access to overcome socio-economic barriers; (iii) diabetes management protocols should explicitly incorporate musculoskeletal health monitoring given the nearly two-fold higher sarcopenia risk in diabetic participants; (iv) care pathways for the 71.4% classified as frail should integrate sarcopenia management alongside falls prevention, nutritional support, and cognitive stimulation; and (v) territorial burden gradients call for differentiated resource allocation formulas within the Canarian health funding distribution rather than per capita allocations that ignore compound Zone 1 vulnerability.

### 4.6. Strengths and Limitations

The strengths of this study include its comprehensive multi-domain assessment battery, reasonable sample size for an island-specific context, use of validated internationally standardised instruments, and integration of economic burden estimation with epidemiological findings. The limitations include the following: (i) SARC-F-based estimates reflect sarcopenia risk rather than confirmed sarcopenia diagnosis—the gold standard of low muscle mass (measured by DXA or BIA) combined with low grip strength or poor physical performance [1] was not available for all participants, and SARC-F has lower sensitivity than diagnostic algorithms. (ii) The cross-sectional design precludes causal inference. (iii) The sample composition—including a high-multimorbidity case group—produces higher overall prevalence than a purely population-representative sample would (users should apply Group 1’s 19.3% as a conservative lower bound for general population planning). (iv) Economic cost estimates involve extrapolation uncertainties and rely on average unit costs that may not reflect island-specific resource utilisation patterns; (v) The study enrolled participants at two contrasting functional levels (without vs. with multimorbidity-related functional decline) without an intermediate group, limiting the characterisation of the full health-functional continuum from healthy ageing to severe functional impairment; future studies should consider a broader stratification of functional status. (vi) Recruitment through primary care attendance may introduce selection bias: individuals attending primary care more frequently are, on average, frailer and more multimorbid than the broader community-dwelling population; the Group 1 estimate (19.3%) should be applied as a conservative lower bound for general community planning. (vii) The SARC-F is entirely self-reported; given that 58.8% of participants exhibited moderate cognitive impairment (Pfeiffer 5–7 errors), responses are subject to information bias through item misunderstanding or recall error. (viii) Key socio-economic confounders—including household income, educational attainment, and access to community services—were not captured in the data-collection protocol; the territorial prevalence gradient likely reflects socio-economic gradients in ageing outcomes, and without individual-level adjustment the diabetes and health-zone ORs may be subject to residual confounding. Future studies should incorporate longitudinal follow-up, objective muscle mass measurement (DXA/BIA), island-specific unit cost surveys, individual-level socio-economic data, and structured caregiver burden assessment.

## 5. Conclusions

This study establishes that sarcopenia risk affects more than one in three community-dwelling older adults in Tenerife, rising to four in five among those with established multimorbidity. It is systematically co-embedded within a nexus of frailty, malnutrition, cognitive impairment, physical inactivity, and functional dependence that collectively define a compound geriatric vulnerability of major public health significance. The estimated annual socio-economic burden of nearly EUR 89 million for Tenerife confirms that sarcopenia is among the most economically costly under-addressed conditions in Spanish ageing policy.

The economic case for prevention is unambiguous: multicomponent exercise–nutrition programmes achieve cost-per-QALY ratios of EUR 3800–7000 against a national threshold of EUR 25,000, with break-even horizons of one to two years. Primary care SARC-F screening—at under EUR 35 per screen—represents one of the most cost-effective health investments available in this population. The territorial concentration of burden in Health Zone 1 calls for place-sensitive, equity-oriented resource allocation within the Canarian health system. These findings underscore the importance of integrating sarcopenia prevention within broader healthy-ageing frameworks for ageing island societies, where the fiscal and demographic stresses of population ageing intersect with structural constraints on public expenditure.

## Figures and Tables

**Table 1:** Socio-demographic and clinical characteristics of study participants, stratified by study group.

Characteristic	Total (*n* = 374)	Group 1—Control (*n* = 274)	Group 3—Case (*n* = 100)	*p*-Value
Age, mean (SD), years	80.4 (4.8)	79.7 (4.5)	82.3 (5.1)	<0.001
Female, *n* (%)	191 (51.1)	149 (54.4)	42 (42.0)	0.045
Male, *n* (%)	183 (48.9)	125 (45.6)	58 (58.0)	—
Diabetes mellitus, *n* (%)	163 (43.6)	—	—	—
Mean BMI (kg/m^2^)	24.7	—	—	—
SARC-F ≥ 4 (at risk), *n* (%)	136 (36.4)	53 (19.3)	83 (83.0)	<0.001

Note: SD = standard deviation; BMI = body mass index; SARC-F = Strength, Assistance, Rise, Climb, Falls; MNA-SF = Mini Nutritional Assessment Short-Form. Secondary clinical characteristics (diabetes prevalence, mean BMI, frailty status, nutritional status, physical activity level, functional dependence, and cognitive status) were assessed at total-sample level only and have been removed from this table following peer-review revision; these characteristics are reported as total-sample frequencies in the text (Section 3.1 and Section 3.3). The “—” in the Male row *p*-value column indicates that the between-group sex comparison applies jointly to the Female/Male rows.

**Table 2:** Sarcopenia risk prevalence stratified by sex, study group, group x sex interaction, diabetes status, health zone, and age group.

Subgroup	*n*	At Risk (SARC-F ≥ 4), *n*	Prevalence (%)	OR (95% CI)
Sex				
Male	183	77	42.1	Reference
Female	191	59	30.9	0.62 (0.40–0.95)
Study Group				
Control (Group 1)	274	53	19.3	Reference
Case (Group 3)	100	83	83.0	20.4 (95% CI 11.2–37.2; *p* < 0.001)
Group × Sex				
Group 1—Male	125	26	20.8	Reference
Group 1—Female	149	27	18.1	0.85 (0.46–1.57)
Group 3—Male	58	51	87.9	28.7 (11.0–74.7)
Group 3—Female	42	32	76.2	15.0 (5.7–39.4)
Diabetes Status				
Non-diabetic	211	63	29.9	Reference
Diabetic	163	73	44.8	1.90 (1.23–2.92)
Health Zone				
Zone 1	81	51	63.0	Highest
Zone 2	119	28	23.5	Lowest
Zone 3	174	57	32.8	Intermediate
Age Group (years)				
71–75	32	11	34.4	Reference
76–80	219	79	36.1	1.07 (0.50–2.30)
81–85	63	24	38.1	1.14 (0.50–2.62)
86–90	46	16	34.8	1.01 (0.41–2.48)
≥91	14	6	42.9	1.40 (0.38–5.15)

Note: OR = odds ratio; SARC-F cut-off ≥4 for positive screen; all ORs are unadjusted; age-group ORs should be interpreted with caution given small cell sizes in the ≥91-year group.

**Table 3:** Estimated annual socio-economic costs of sarcopenia in Tenerife and Spain (2023 EUR prices).

Cost Component	Per Patient/Year (EUR)	Tenerife (EUR M)	Spain National (EUR M)	Source
Direct Costs				
Primary care and outpatient	1200	3.1	420	[10]
Hospital admissions (falls/fractures)	3800	9.8	1330	[22]
Rehabilitation and physiotherapy	950	2.4	333	[9]
Nutritional supplementation	720	1.9	252	Conservative estimate
Long-term care/institutionalisation	12,000	31.0	4200	[22]
Indirect Costs				
Informal care/caregiver burden	8500	21.9	2980	[7]
Lost caregiver productivity	3200	8.2	1120	Estimated
Premature mortality (DALY)	4100	10.6	1440	WHO approach
Total Annual Burden	34,470	88.9	12,075	—
Preventable burden (60% reduction)	20,680	53.3	7245	[25]

**Table 4:** Cost-effectiveness of sarcopenia prevention and treatment interventions, ranked by cost per QALY gained.

Intervention	Cost/Patient/Year (EUR)	Efficacy (Risk Reduction)	Cost per QALY (EUR)	Break-Even (Years)	Grade
SARC-F screening (primary care)	18–35/screen	Early detection	1200–2800	<1	A
Combined exercise + nutrition	900–1400	35–55%	3800–7000	1–2	A
Resistance/progressive exercise	480–820	25–40%	4200–8500	2–3	A
Protein supplementation (≥1.2 g/kg/day)	290–550	15–28%	5100–9200	2–4	A
Community falls prevention	350–600	20–35% falls down	4800–9500	2–3	B
Vitamin D supplementation	80–150	10–18%	6500–14,000	3–5	B
Polypharmacy review	120–200	10–15%	7200–12,000	3–4	C
Digital/telehealth monitoring	600–1100	15–25%	8000–16,000	3–5	C
Spanish cost-effectiveness threshold	—	—	≤25,000	—	—

Note: QALY = quality-adjusted life year. Grade: A = strong cost-effectiveness (≤EUR 10,000/QALY); B = moderate (EUR 10,001–20,000); C = acceptable (EUR 20,001–25,000). Efficacy estimates from published meta-analyses [11,12,25,26,27].

## Data Availability

The data presented in this study are available on request from the corresponding author. The data are not publicly available due to privacy restrictions and applicable data protection legislation.
